# Effect of urban structure, population density and proximity to contagion on COVID-19 infections during the SARS-CoV-2 Alpha and Omicron waves in Málaga, Spain, March 2020 to December 2021

**DOI:** 10.2807/1560-7917.ES.2025.30.3.2400174

**Published:** 2025-01-23

**Authors:** Sebastián Alejandro Vargas Molina, Juan Francisco Sortino Barrionuevo, María Jesús Perles Roselló

**Affiliations:** 1 University of Málaga, Málaga, Spain

**Keywords:** covid-19, spatial distribution, urban, point pattern analysis

## Abstract

**Background:**

The potential impact of urban structure, as population density and proximity to essential facilities, on spatial variability of infectious disease cases remains underexplored.

**Aim:**

To analyse the spatial variation of COVID-19 case intensity in relation to population density and distance from urban facilities (as potential contagion hubs), by comparing Alpha and Omicron wave data representing periods of both enacted and lifted non-pharmaceutical interventions (NPIs) in Málaga.

**Methods:**

Using spatial point pattern analysis, we examined COVID-19 cases in relation to population density, distance from hospitals, health centres, schools, markets, shopping malls, sports centres and nursing homes by non-parametric estimation of relative intensity dependence on these covariates. For statistical significance and effect size, we performed Berman *Z*1 tests and Areas Under Curves (AUC) for Receiver Operating Characteristic (ROC) curves.

**Results:**

After accounting for population density, relative intensity of COVID-19 remained consistent in relation to distance from urban facilities across waves. Although non-parametric estimations of the relative intensity of cases showed fluctuations with distance from facilities, Berman’s Z1 tests were significant for health centres only (p < 0.032) when compared with complete spatial randomness. The AUC of ROC curves for population density was above 0.75 and ca 0.6 for all urban facilities.

**Conclusion:**

Results reflect the difficulty in assessing facilities’ effect in propagating infectious disease, particularly in compact cities. Lack of evidence directly linking higher case intensity to proximity to urban facilities shows the need to clarify the role of urban structure and planning in shaping the spatial distribution of epidemics within cities.

**Key public health message**

**What did you want to address in this study and why?**

Cities are recognised as places of interest in public health and epidemiology. Aspects such as urban structure and planning, reflected for example in population density as well as supply and distribution of urban facilities, have been overlooked but could play an important role in determining spatial distribution of an infectious disease. We assessed the role of these co-variates on the spatial pattern of COVID-19 cases in Málaga, Spain.

**What have we learnt from this study?**

Once controlling in our analysis for population density as main factor for positive COVID-19 cases, we found that proximity to most urban facilities, that we considered to be contagion hubs, had a small effect on the recorded number of infections. This small-scale effect was similar when there were mitigation measures such as lockdowns (Alpha wave) in place or when they were lifted (Omicron wave).

**What are the implications of your findings for public health?**

Málaga’s compact urban structure with close proximity between densely populated areas and urban facilities likely nullified any impact on relative intensity of cases. We suggest applying a geospatial perspective merging urban studies with public health considerations to evaluate whether, and how, distance to and spatial distribution of exposure factors may affect spatial distribution of infectious diseases in urban areas and in similar settings.

## Introduction

COVID-19 emerged in Europe in early 2020 as a highly contagious disease with a swift and extensive spread, particularly in urban settings. Given that more than half the world’s population live in cities [1-3] with a high concentration of people and activities, it can be inferred that close proximity contributed to the rapid spread of the virus. In Europe, the COVID-19 pandemic was most pronounced in Spain and Italy during its early stages [4].

Governments worldwide enacted non-pharmaceutical interventions (NPIs) to mitigate the spread of the pandemic, and Spain was no different in this regard [5]. Mobility-restricting measures commonly referred to as lockdowns were one of the main NPIs [6], with variable degrees of reach and effectiveness [7-9]. Despite variations within adopted NPIs worldwide, including differences in duration and type of activities or services halted, a shared factor was the closure or restriction of essential urban public facilities and services [10], i.e. the infrastructure providing the population of an area with goods and public services [11]. These services encompassed educational, economic, health and leisure activities and facilities, and measures temporarily altered the urban landscape. In retrospect, this allows us to study how changes in the urban environment impact the progression of an infectious disease.

Studies looking at the effect of urban settings on the spread of COVID-19 have focused on two main aspects: (i) the modelling of cases as a function of the environmental and/or economic variables which are labelled as ‘urban’, or (ii) the description of the spatial patterns of outbreaks at different points in time during the pandemic [12-15].

The first involves using linear regression models, such as geographically weighted regression, to predict case numbers based on relevant urban variables such as population density, income, unemployment, mobility and access to facilities. These studies aggregate cities into administrative areas, treating them as rows in a data matrix, and highlight the contribution of vulnerability and exposure factors to case increases. However, issues such as multicollinearity among variables often lead to overfitted models and self-reinforcing assumptions, limiting the reliability of these findings [12-15].

The second approach utilises spatial statistics to provide more detailed insights into interurban variability. Techniques such as local spatial autocorrelation and hotspot detection often rely on data aggregated at varying administrative boundaries, which introduces the modifiable areal unit problem (MAUP), affecting results [16-19]. To mitigate this, some studies employ spatial point pattern methods to create continuous surfaces of relative case intensity, also known as relative risk (RR) [20], as the spatial equivalent of the incidence rate in epidemiology. However, while incidence is a time-bound measurement (cases over person-time unit) [21], in point pattern analysis the RR is a spatial measurement of cases relative to population, per unit area [22].

For the specific case of the city of Málaga, Spain, research has focused on two main fronts: (i) data collection and management and characterisation of the initial spatial trends of the severe acute respiratory syndrome coronavirus 2 (SARS-CoV-2) Alpha variant (Phylogenetic Assignment of Named Global Outbreak (Pango) lineage designation (B.1.1.7) wave [23], and (ii) methods for the quantification and cartographic representation of risk in general [23-25]. While these efforts provide the main precedent for the present investigation, there is room for further research, particularly in relation to population density and distance to contagion hubs, since the role of population density as the main factor driving case intensity during an epidemic remains inconsistent in the literature due to data availability and methodological shortcomings [26].

We thus propose focus on two specific factors: (i) case distance from urban facilities and (ii) cases in relation to population density. While these factors are inevitably intertwined, we consider the latter as the main confounding variable.

This study aims to investigate the spatial patterns of COVID-19 case intensity and relative intensity during two distinct pandemic waves – the Alpha wave representing a period of restricted mobility (lockdowns), and the Omicron (Pango lineage B.1.1.529) wave representing no lockdown. This comparison allows us to examine the role that distance to urban facilities might play in shaping the spatial pattern of relative intensity, which may not be related to the effectiveness of NPIs in slowing the spread of the disease.

Specifically, we seek to determine whether the relative intensity of cases during the Alpha wave remained constant and proportional to population density, and whether the relative intensity of cases during the Omicron wave increased as proximity to urban facilities decreased, corresponding to greater interpersonal interactions in the absence of lockdown measures.

## Methods

### Setting

The study used data collected between February and March 2020 and between December 2021 and March 2022 in Málaga, the sixth largest city in Spain, with a population of 579,076 residents, and a population density of ca 12,000 inhabitants per squared kilometre in its urban zone. Located within the municipality of the same name in the autonomous community of Andalucía, Málaga is a prominent tourism hub in the southern coast of Spain (*Costa del Sol*), receiving a high number of visitors annually.

**Figure 1:**
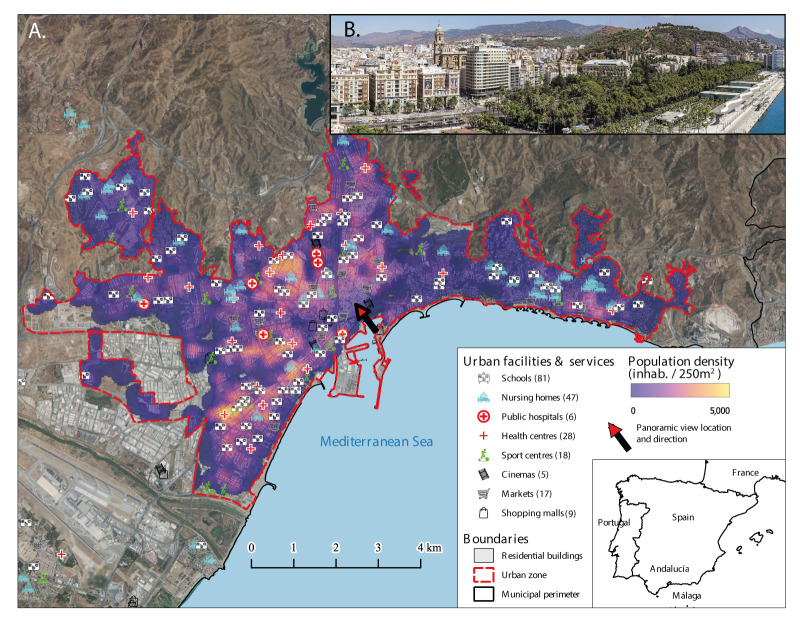
Study area including (A) general map and aspects of interest, and (B) panoramic view of the city, taken from downtown area at the port, and directed at the west, Málaga urban zone, Spain

Source: own elaboration with data from Andalucía Census Bureau and the Málaga Municipality Open Data Platform. Panoramic view picture taken from the Málaga Municipality website. Orthophoto from 2019 by the Andalucia Census Bureau.

This research specifically centred on the urban area within the municipality, with a focus on examining the spatial variability of population density and the placement of urban facilities and amenities, as depicted in Figure 1. We selected these urban facilities for the specific roles they play in day-to-day urban life, encompassing schools (both public and private), markets, health centres, public hospitals, cinemas, shopping malls, sport centres and nursing homes (all of them private, except one).

In terms of urban structure, the city has been characterised in the framework of the ‘Mediterranean city model’ [27] and the ‘compact city’ model [28]. These models of urban planning and design have been considered the urban corelate of sustainable development for the past 30 years, aimed at achieving a certain level of population density, mixed land use and compactness to encourage social interaction, energy efficiency, pollution reduction and economic development [28-30].

Regardless of if or how Málaga deviates from these models the city exhibits some characteristics of these models, particularly in its housing type, with a prominence of apartment buildings [31] in zones mixed with different activities and establishments. Most facilities are within a ca 2 km radius from any residential dwelling.

As regards to the development of the pandemic in the city, while the Alpha wave (occurred February–March 2020) was characterised by the predominance of NPIs due to the unavailability of vaccines, the Omicron wave (December 2021–March 2022) occurred when lockdowns had been lifted and vaccinations were available. The widespread removal of lockdown measures occurred in May 2021 [32], approximately 5 months before Omicron cases were recorded. During this period, mobility and activities were unrestricted, allowing for the analysis of ‘imprinted’ patterns of the free circulation of an infectious disease in relation to key aspects of cities that were previously closed or restricted as part of the mitigation strategies.

### Data

We obtained data on COVID-19 cases from the Málaga Regional Hospital, which centralised records of all SARS-CoV-2 tests performed at any testing centre or facility within the municipality. The medical records of every tested person, detailing the type of test applied and its results, was provided by the Málaga Delegation of the Health Office, *Junta of Andalucía* and is protected under the Spanish Organic Law on Protection of Personal Data, with each patient’s address serving as the sole personal identification value. As such, data were provided with no demographic variables such as sex, occupation, age or ethnicity.

The addresses of individuals who tested positive for any type of molecular (PCR / TMA) or serological (IgG / IgM) test underwent geocoding, which rendered the spatial point dataset. In adherence to privacy protection laws, public sharing of these data is not allowed, which hinders the reproducibility of this specific analysis.

Urban infrastructure and services data, in the form of spatial layers, was obtained from the Málaga Municipality Open Data Platform (https://datosabiertos.malaga.eu). For this study, we used point data in shapefile format representing the location of different types of urban facilities. Spatial layers contained no data on the number or the characteristics of visitors.

An important caveat to consider is that the data obtained during each wave display notable differences in both consistency and quality, as testing availability and adoption increased throughout the pandemic. For instance, records during the Alpha wave were available for cases in March 2020 only (n = 129). During the Omicron wave (December 2021–March 2022) 29,919 cases were recorded. Therefore, while we used the whole Omicron dataset to explore the intensity of cases across weeks, to make the comparison between waves meaningful we randomly sampled 129 cases from the whole Omicron wave dataset.

### Software

Excel 2016 was used for basic data preprocessing, including column naming and deletion of records that were duplicated, outside of Málaga or with no recorded address, as the original datasets were provided in spreadsheet format by the Málaga Delegation of the Health Office.

Geoprocessing was made in a variety of Geographic Information Systems (GIS) applications. The ArcGIS geocoding toolbox within the ArcGIS Pro platform [33] was used for georeferencing addresses, which provided the most accurate application programming interface (API) service to automate georeferencing of the text addresses included in the original datasets. Google Earth Pro version 7.3.6.9345 was used to correct imprecise geocoding caused by inconsistencies between addresses and postcode areas. The software QGIS version 3.22.9 Białowieża [34] was used for cartographic design and general shapefile processing, given its open-source nature and high capabilities for integrating different file formats.

Statistical analysis was performed using R Language version 3.6.3 in the R Studio IDE version 2023.06.1 + 524 (Posit, Boston, United States). The package *spatstats* version 2.1–0 was used for this task since it is specifically designed for spatial point patter analysis.

### Spatial point pattern analysis

We performed a spatial point pattern analysis to study the pattern of reported positive cases [22,35,36]. We used the address of cases with a COVID-19 positive test result within the urban area of Málaga, during (i) the Alpha wave (n = 129 cases) and (ii) Omicron wave (n = 29,919 cases). We subdivided the latter by week, from week 48 2021 to week 12 2022. We used case intensity (events per unit area) as first order effect within the domain of spatial point pattern analysis.

We explored variations in local intensity of positive COVID-19 cases across the study area by kernel density estimation (KDE). Kernel radius was set at 300 m^2^ based on Nearest Neighbour Analysis on the location of cases.

However, the number of cases is often a reflection of the underlying population density, in this context being point data of at-risk population referred to as controls. As such, to analyse the relationship of the intensity and interaction of cases with other covariates, we compared the number of cases to the underlying population at risk, which can be seen in more detail in the Supplementary Material. The control point data set is a simulated point pattern for controls proportional to the total population in each census tract [35,37]. In this case, a regular polygon from a 250 m^2^ grid containing census-level data created by the Andalucia Census Bureau [38].

To evaluate whether the varying intensity of cases could be explained by distance between case location and the different types of urban facilities, we calculated a nonparametric estimation of case intensity as a function of distance from urban facilities as the main covariate.

We estimated a baseline relative intensity of the point pattern for each urban facility based on a given covariate [22]. The baseline is a fitted point process model of cases compared to population density of controls, for which intensity variation was evaluated as a function of the distance from urban facilities. For this step, we randomly sampled 129 cases from the Omicron wave dataset to make the comparison against the Alpha wave data meaningful.

We performed Berman's Z tests for point process model to test against the null hypothesis of complete spatial randomness (CSR) in relation to distance from urban facilities, and receiver operating characteristic (ROC) curve tests, as well as the area under the curves (AUC) to assess the statistical significance of the estimated relative intensity as a function of distance from several urban facilities and measured the effect size of each covariate. We also compared the relative intensity to population density as the main confounding variable.

We used nonparametric estimations [20], which can be useful for studying a practical, real-world problems [36] such as public health phenomena. Technical and formal details are expanded in the Supplementary Material.

## Results

**Figure 2:**
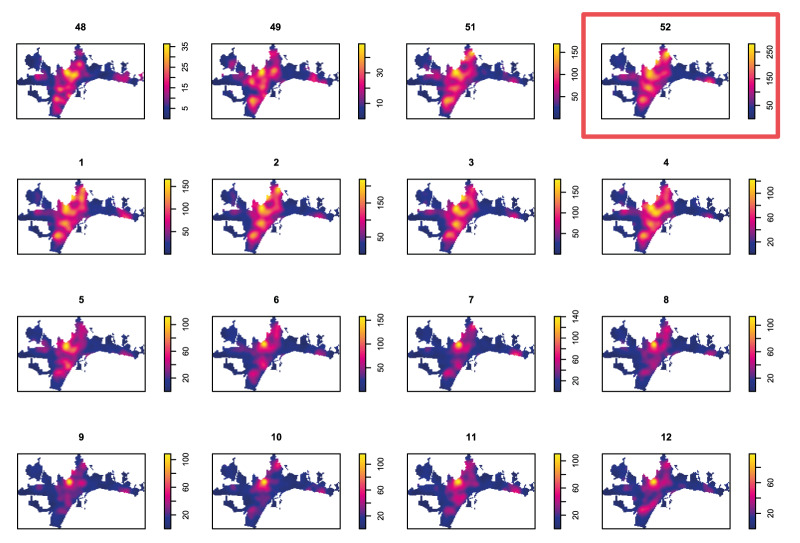
COVID-19 case intensity by week, Málaga, Spain, December 2021–March 2022

The highest case count (n = 3,891) and density, with up to 350 cases per 300 m^2^ (Figure 2, enclosed in red box), was recorded during week 52 2021 (December), which represents the selected bandwidth parameter for the KDE. While visual inspection alone is insufficient, the spatial pattern appeared similar across all the weeks of the Omicron wave, varying primarily in intensity. The overall ‘imprinted’ pattern resembles that of the population density (Figure 1).

Values of intensity bar vary according to the intensity estimated for each week. Scalebar represents number of cases per 300 m^2^.

**Figure 3:**
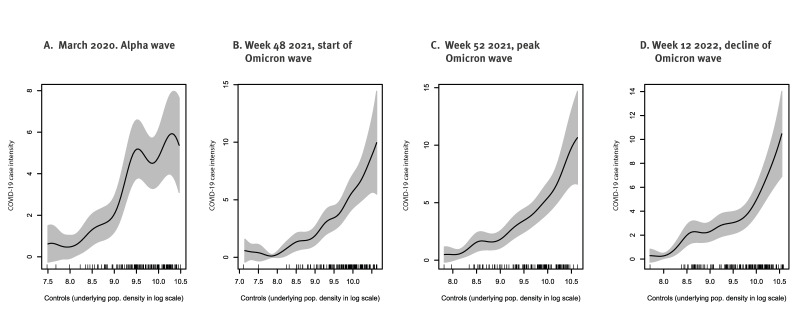
Nonparametric estimation of COVID-19 case intensity as a function of the at-risk population during (A) the Alpha wave, (B) the start of the Omicron wave, (C) the peak of the Omicron wave and (D) the decline of the Omicron wave, Málaga, Spain, March 2020 and December 2021–March 2022

We report a positive relationship between case intensity and population density within the urban area of Málaga (Figure 3). This pattern is consistent when comparing the Alpha wave with different key weeks during the Omicron wave (week 48 2021, week 52 2021 and week 12 2022). However, fluctuations are noted, which could be explained by other factors, such as proximity to urban facilities or contagion hubs.

Gray shading represents 95% confidence intervals at 95%.

For each selected week from the Omicron wave, 129 cases were randomly selected and compared to the 129 cases available for the Alpha wave.

**Figure 4:**
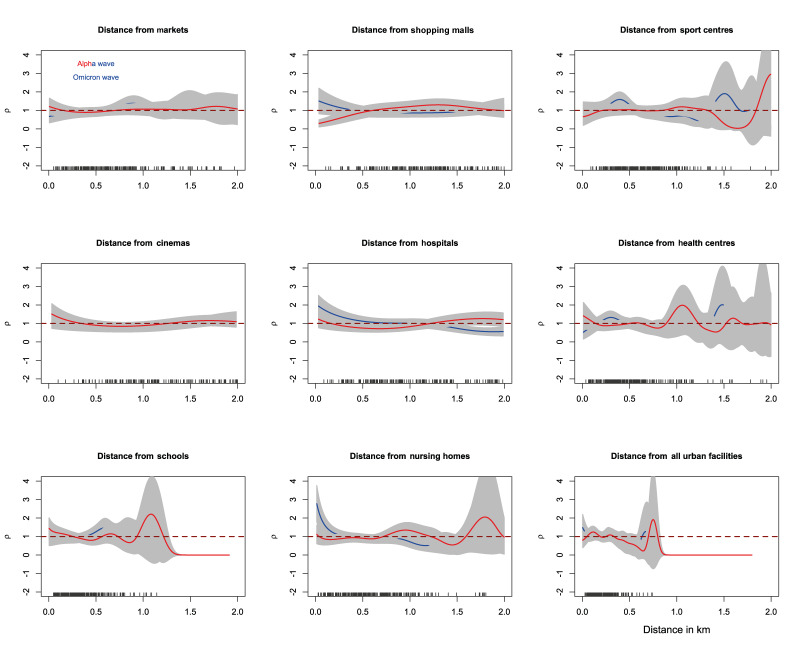
Nonparametric estimation of relative intensity of COVID-19 cases as a function of distance from different urban facilities, Málaga, Spain, during the Alpha (March 2020), and Omicron (December 2021–March 2022) waves

Having established the close relationship between case intensity and population at-risk during the Alpha and Omicron waves, we estimated the relative intensity of cases as a function of distance from urban facilities during both the Alpha and Omicron waves (Figure 4). In this way, we assessed the specific effect of this covariate with minimal contamination from the effect of population density, given its strong correlation with case intensity.

The null model is indicated by a value of 1 (red dashed line). At this baseline, intensity of cases remains proportional to the underlying population at risk. Fluctuations of the function above or below this baseline are relative intensity variations attributed to the distance from the indicated urban facility. All axes display the same margin values for comparison purposes. Gray shading represents confidence intervals at 95%.

For both COVID-19 waves, the relative intensity of cases remained similar regardless of distance from markets, shopping malls and cinemas, which also are the least abundant urban facilities (Figure 1). On the other hand, for sport centres, hospitals, health centres and nursing homes, the relative intensity of cases increased as distance from these locations reduced (and vice versa). This trend was also noted when taking into account all urban facilities.

Interestingly, this trend was similar during both waves, despite the first wave characterised by a generalised lockdown and the second wave by a return to regular public life, except for Health centres. Relative intensity of cases at a distance of ca 100m from health centres increased during the Alpha wave (ca 1.5), and diminished during the Omicron wave (ca 0.5). Comparison between all urban facilities suggests that distance had a negligible effect on the spatial relative intensity of cases, once accounting for population density.

**Figure 5:**
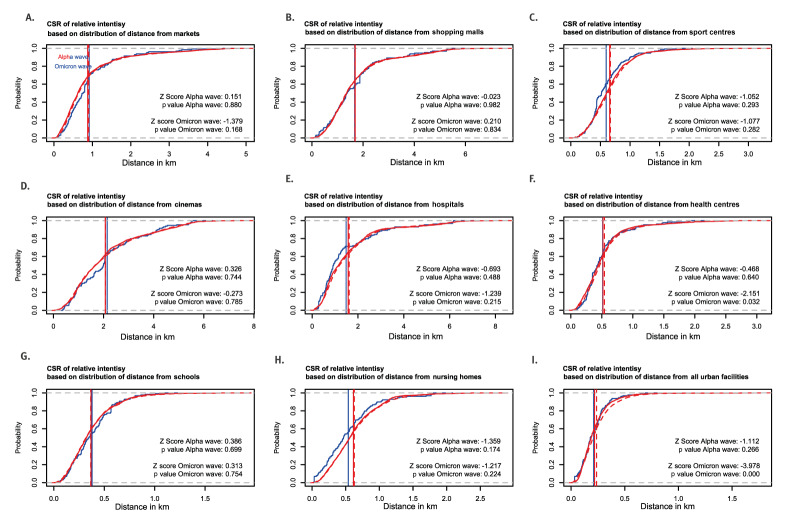
Berman *Z*1 test for complete spatial randomness of relative intensity of COVID-19 cases as a function of distance from urban facilities, Málaga, Spain, during the Alpha (March 2020) and Omicron (December 2021–March 2022) waves

Fluctuations in relative intensity as a function of distance from health centres were of statistical significance for the Omicron wave only (p value < 0.032) when compared with CSR (Figure 5).

Vertical solid lines represent the mean of the observed distribution of values of the spatial covariate (distance from urban facilities) at data points for Alpha and Omicron waves. Vertical dashed line represents the mean of the null model [22]. Z-score and p values are provided within each plot for each wave.

The relative intensity of cases as a function of distance from urban facilities did not significantly differ from CSR during the Alpha wave. However, for the Omicron wave, relative intensity of cases increased slightly near Health centres, and when taking into account all urban facilities, beyond what would have been expected for CSR.

**Figure 6:**
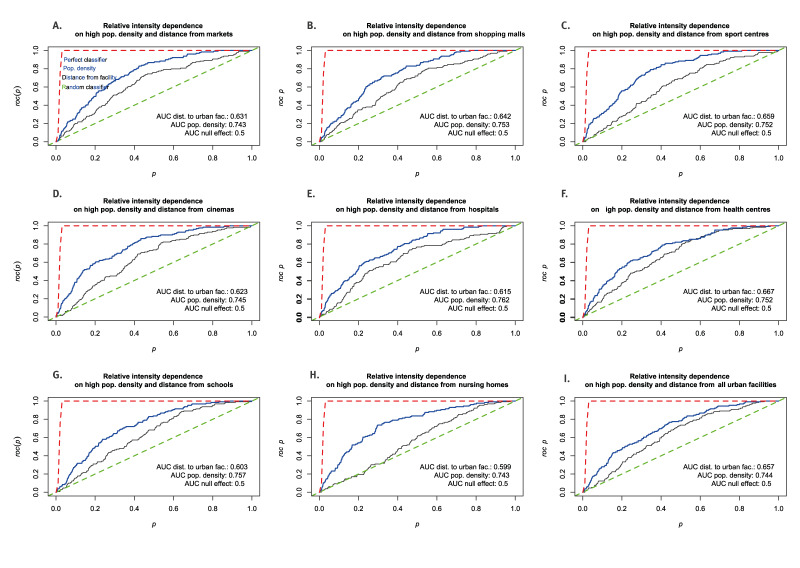
Comparison of receiver operating characteristic (ROC) curves and area under the curve (AUC) tests for the effect of high population density vs distance from different urban facilities on COVID-19 case intensity, Málaga, Spain, during the Alpha (March 2020) and Omicron (December 2021–March 2022) waves

For the effect size on relative intensity of cases, in general, population density has double discriminatory power for classifying the study region in high and low intensity areas when compared with the distance from urban facilities. This is measured by the area under the curve (AUC), while also noting that both ROC curves, one for each covariate, follow each other in a similar fashion (Figure 6), indicating the close spatial relationship between population density and urban facilities.

ROC is a plot showing the ability of the covariate to separate the study region into areas of high and low density of cases. The closer the covariate curves (black and blue solid lines) are to the perfect classifier (red dashed line), the more these covariates explain the separation of high- and low-density zones in the region. Conversely, the closer they are to the random classifier (green dashed line), the less effect they have on said separation. For a point pattern and a given covariate, the AUC is a numerical index that measures the ability of the covariate to separate the spatial domain into areas of high and low density of points. As such, an AUC = 0.5 would mean a random classifying effect (green dashed line).

In urban planning terms, this may be a consequence of the compact urbanisation model of Málaga mentioned earlier, given that within the main urban zone, facilities are no more than ca 2 km from housing dwellings (Figure 1), which are, in turn, blocks of apartment buildings. Thus, dwellings and facilities in the city overlap, which seem to neglect any effect distance from urban facilities could otherwise play in the recorded intensity of cases.

## Discussion

Based on our results, and contrary to our starting hypotheses, we were unable to find compelling evidence of the role of proximity to urban facilities, as hubs of potential contagion, on the relative intensity of COVID-19 cases during the Alpha and Omicron waves. Instead, population density, our main confounding variable, was positively correlated with case intensity during both waves. Even when comparing facilities, differing in spatial distribution and number, the effect on relative intensity remained similar between periods of enacted and lifted NPI.

Our results are not in agreement with previous studies addressing the main factors of interest [12,13,15,37]. In Wuhan [12], the availability of schools and markets was positively associated with higher incidence rates of COVID-19, while population density negatively correlated with incidence rate. Similarly, in Tokyo, higher incidence rates at municipality level were linked to the availability of various urban facilities and negatively correlated with household crowding [13]. Indirectly, previous research in Málaga associated accessibility (measured as an index built from unspecified proximity variables) with higher incidence rates [15]. The study using the most similar methodology to ours suggested that areas of Neukölln in Berlin with higher relative intensity of COVID-19 cases were those with shorter proximity to places of social interaction and higher population density per building [37].

In contrast, our results show that for Málaga, population density has greater explanatory power over the relative intensity of cases, effectively nullifying any effect of the availability and distance from urban facilities. This finding remained consistent when comparing scenarios of enacted and lifted lockdown measures. However, the inconsistencies between our results and those of previous research may be attributed to the diversity in methods, data and scale in the summarised research, or to the particularities of each analysed city.

While it is not possible to generalise our results to any other city, we suggest that it is necessary to thoroughly discuss the role of urban planning on public health. This includes not only basic and well-stablished (but not necessarily well-achieved) areas like health services and public sanitation, but also overlooked areas such as housing, zoning and non-health / medical-related urban facilities. Currently, research on this topic, this study included, exhibits high variability in results, methods and data spatial and temporal resolution.

On the limitations of this study, first, due to data constraints, we were unable to account for variations in supply, capacity, transportation and attractiveness between different types of facilities, as well as in the variability of sanitary measures taken in each type of facility. For instance, markets outnumbered hospitals, with the former typically having smaller capacity and strict sanitary protocols when compared with the any other type of facility. Survey designs, big data approaches, and urban analytics could offer ways to overcome such constraints, showing nuanced insights into the attractiveness of different urban facilities, and serving as a new covariate in future studies.

Second, given the nonparametric framework of the spatial point pattern analysis, it was not possible to provide formal statistical evidence that allows for the extrapolation of these results to any other city, which limits the external validity of the results to the city of Málaga only. However, it provides some assumptions and hypotheses about how the intensity of cases for similar cities, with similar population density and compact urban structures, could vary on the interurban scale. This could be particularly relevant for cities like Valencia, Sevilla, Barcelona, Turin, Rome, Geneva and Thessalonica, which, along with Málaga, were recognised as examples of the "Mediterranean city model", a sustainable urban planning approach that emphasizes compactness, walkability, and mixed land use [27]. While such a structure promotes higher accessibility to health services and sanitation due to close proximity, the mixed land uses closely overlapped with densely spaced residential dwellings across a city could promote a generalised spread of an infectious disease such as COVID-19.

Third, the reproducibility of this specific analysis is not possible due to the legal protection of personal data, presented in the form of geolocated addresses for cases positive for COVID-19. However, our study provides a reproducible design which can serve as a template for analysing similar data from other locations.

## Conclusion

With the current study’s results and acknowledged limitations, different types of research, aside from observational studies, whether spatial or not, are important. Exploring quasi-experimental and simulation-based designs would significantly enhance the depth of our understanding regarding the role of urban factors in both intensifying and mitigating the spread of infectious disease. Further investigations along these proposed lines, while not exhaustive, could contribute to a better understanding how epidemics evolve in urban contexts. It is important to consider that urban areas constitute the environment where a growing share of the world’s population is living. Consequently, they can be expected to play a role as epicentres in future health crises triggered by infectious diseases. How we configure, manage and design cities needs thus to be considered a key aspect in the public health domain.

## Supplementary Data

656000 Supplementary Material
