# Supplementary material for: Effect of urban structure, population density and proximity to contagion on COVID-19 infections during the SARS-CoV-2 Alpha and Omicron waves in Málaga, Spain, March 2020 to December 2021
Source: Euro Surveill. 2025 Jan 23;30(3):2400174. doi: 10.2807/1560-7917.ES.2025.30.3.2400174 (PMC11914961; doi:10.2807/1560-7917.ES.2025.30.3.2400174)
Supplement: Supplementary Material [file 24-00174_VARGAS-MOLINA_Supplement.pdf]

Supplementary material

Varying intensity of disease cases as a function of spatial co-variates

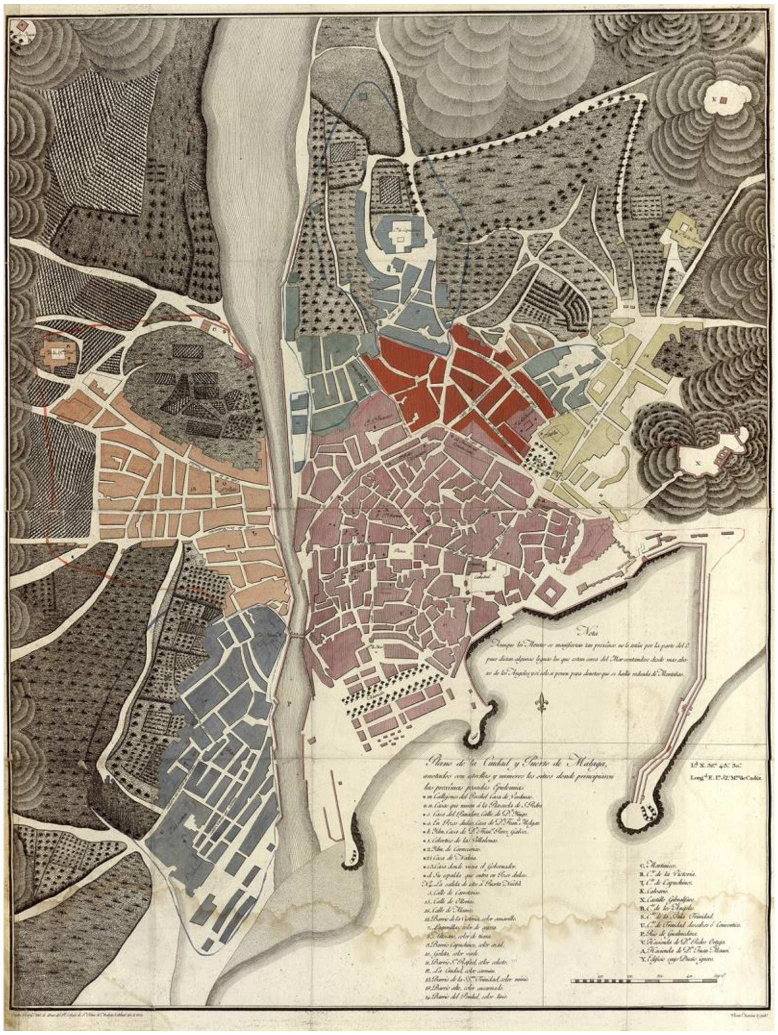

Map of Yellow Fever in Malaga City, 1800.

“This supplementary material is hosted by *Eurosurveillance* as supporting information alongside the article”Effect of urban structure, population density and proximity to contagion on COVID-19 infections during the SARS-CoV-2 Alpha and Omicron waves in Malaga, Spain, March 2020 to December 2021”, on behalf of the authors, who remain responsible for the accuracy and appropriateness of the content. The same standards for ethics, copyright, attributions and permissions as for the article apply. Supplements are not edited by *Eurosurveillance* and the journal is not responsible for the maintenance of any links or email addresses provided therein.”

Spatial point pattern analysis. Technical aspects

A point pattern is a dataset consisting of a set of locations in a defined study region, at which events of interest have been recorded. The location of these events is represented as points within the specified region. This can be formally represented as a set (Gatrell et al. 1996a; Baddeley, Rubak, and Turner 2015; González and Moraga 2023a). For this collection of event locations, the interest is to describe their intensity (events per unit area) and interaction (distance between events), which are termed as first order and second order effects, respectively, within the domain of spatial point pattern analysis.

Specifically for this paper, we are interested in first order effects of X, which represents a collection of addresses of people with a COVID-19 positive test result within W, the urban area of Malaga, corresponding to both, Alpha wave (129) and Omicron wave (29,919). The latter is subdivided by week (WK), from WK 48, 2021 to WK 12, 2022.

Local intensity across the study area can be represented by a kernel density estimation (KDE). The formal development has been discussed plenty by mathematicians and statisticians (Moraga 2019; Baddeley, Rubak, and Turner 2016) but in practical terms, it can be defined as $\hat{\lambda}_u = \frac{\#(S \in C(u,r))}{\pi r^2}$, where S are points within $C(u, r)$, a circle of radius r centered at the location of interest u (O'Sullivan and Unwin 2010). A critical point for this measurement is the "appropriate" radius of the kernel (a bandwidth in technical terms), which was set at 300 square meters, based on Nearest Neighbor Analysis (refer to the code routine).

In this case, the immediate interest would be to calculate the KDE of positive COVID-19 cases. However, the number of cases is often a reflection of the underlying population density, in this context being point data of at-risk population to as controls. As such, to analyze in any meaningful way the relationship of the intensity and interaction of cases with other co-variates, first it is needed to compare the amount cases by the underlying population at risk.

In spatial point pattern analysis, the relative risk RR can be estimated by a KDE ratio which provides a measure of how the intensity of cases λ_{cases} compares to the intensity of controls $\lambda_{\text{controls}}$ at each location u in the study area W, helping to identify areas with a higher or lower risk of COVID-19 incidence. Formally, it can be represented as $\text{RR}(u) = \frac{\lambda_{\text{cases}}(u)}{\lambda_{\text{controls}}(u)}$. The control point data set is a simulated point pattern for controls proportional to the total population in each tract (Gatrell et al. 1996b) (Lambio et al. 2023); in this case, a regular polygon from a 250 square meters grid containing census-level data created by the Andalucia Census Bureau.

Then, the main objective is to evaluate if the varying intensity $\hat{\lambda}_u$ of the given point pattern X can be potentially explained by a set of other co-variates Z measured at every location u of the whole study region W. Formally $\hat{\lambda}_u = \rho(Z(u))$, where ρ is the function that describes how the intensity varies for different values of $Z(u)$ (Baddeley

et al. 2012). For this specific case, we are interested in population density $Z_1(u)$ and distance from different types of urban facilities $Z_2(u)$, as co-variates for the intensity of cases.

However, bringing back the concern for the relative risk RR mentioned above, the intensity of the process based on a given co-variate needs to be assessed from a given baseline estimated at each location $B(u)$, which is a relative intensity, formally represented as $\hat{\lambda}_u = \rho\big(Z(u)\big)B(u)$ (Baddeley et al. 2012). In this case, the baseline is a fitted point process model of cases against the density of controls, for which intensity variation is evaluated as a function of the distance from urban facilities. It is worth mentioning that these are non-parametric estimations (Baddeley et al. 2012), which can be useful for studying practical, real-world problems such as public health phenomena (González and Moraga 2023b).

Dr. Pavel Chernavskiy and Dr. Luc Anselin have excellent online courses on the subject:

Course on Spatial Statistics by Dr. Pavel Chernyavskiy

Course on Spatial Cluster Analysis by Dr. Luc Anselin

Data requirements

The following coding routine is designed to function as a blueprint for replicating the same analysis in any given city, contingent upon the availability of requisite data in a specific format:

1. A polygonal shapefile layer delineating the study area, typically representing the perimeter of any urban zone under investigation.
2. Either a point data shapefile or a CSV file containing coordinates pinpointing the locations of individuals who have tested positive for the disease under scrutiny.
3. Either a point data shapefile or a CSV file containing coordinates marking the locations of the population at risk. This dataset could consist of individuals who tested negative for the disease of interest, albeit serving as a sample of the overall population at risk. Alternatively, this dataset can be simulated through random points based on census tract data.
4. Either a point data shapefile or a CSV file containing coordinates for the locations of various urban facilities of distinct types.

With the exception of point location cases, all requisite data can typically be sourced from public open data repositories.

Note on control data (population at-risk)

Since there is no dataset providing the actual address of each individual in any given city, there are three options to estimate population at risk, each one with their own set of advantages and disadvantages.

1. Georreferencing address of people with a negative test result.
    a. While this option is more accurate to represent the real location of the population at risk, the comparison would be made against a sample of the population.
    b. It could be more costly due to the need of georreferencing services, like Google or ESRI API's.
2. Using a weighted KDE estimation of the centroid of each census tracts polygon with total population data.
    a. This option allows to consider the whole population at risk, but in an aggregated manner. Hence, accuracy about the real spatial distribution of the underlying population depends on the location of the centroid of the polygon.
    b. This is an easy and straightforward operation, and can be done with any mainstream GIS desktop software (QGIS or ArcGIS).
3. Based on census tracts population data, simulate point controls proportional to the population in each tract.
    a. This procedure can be considered a middle point between the previous options, since the location of population at risk can be simulated by creating a random set of points within each polygon, amounting to a fraction or the total population in the given tract. However, this assumes unrealistic locations, which would need to be manually adjusted in order to not represent locations at vacant land.
    b. As the previous option, this is an easy and straightforward operation, and can be done with any mainstream GIS desktop software (QGIS or ArcGIS).

Study area and collection of points

First, an observation window is created. This will be the study region within which the spatial point pattern is analyzed. For simplicity sake, we will assume a rectangular window, representing a fictional urban area.

Next, a set of artificial clustered point patterns (Matern II process) is created to represent the addresses of both cases and controls for a given disease, as well as for points representing the locations of urban facilities.

Controls are more numerous than cases and urban facilities.

```
# Create observation window, representing a fictional urban area
W <- owin(c(10,20), c(10,20), unitname=c("kilometer"))
# Create and plot spatial point patterns
par(mfrow = c(1,3))
# People tested positive for an infectious disease
X_cases <- rMatClust(2, 0.5, 1.5, win = W)
plot(X_cases, cols = "red", main="Positive cases of disease X")
# People tested negative for an infectious disease
X_controls <- rMatClust(4, 0.5, 1.5, win = W)
plot(X_controls, cols = "blue", main="Controls (population at risk")
# Urban facilities (i.e., supermarkets, schools, health centers)
X_facilities <- rMatClust(0.3, 0.5, 2, win = W)
plot(X_facilities, main="Urban facilities")
```

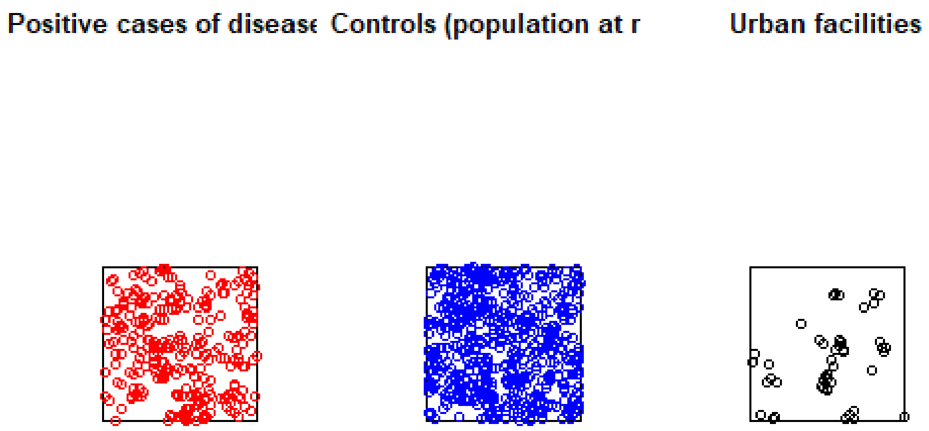

First order effect analysis

Next, images for the density of cases and controls, and for the distance raster from urban facilities are created.

```
par(mfrow = c(1,3))

# Density of cases with automatically selected bandwidth

D_cases <- (density(X_cases, bw.ppl))
plot(D_cases, main="Density of positive cases")

# Density of controls with automatically selected bandwidth

D_controls <- (density(X_controls, bw.ppl))
plot(D_controls, main="Density of controls")

# Distance raster to facilities

X_facilities_dist <- distmap(X_facilities)
plot(X_facilities_dist, main="Distance from urban facilities")
```

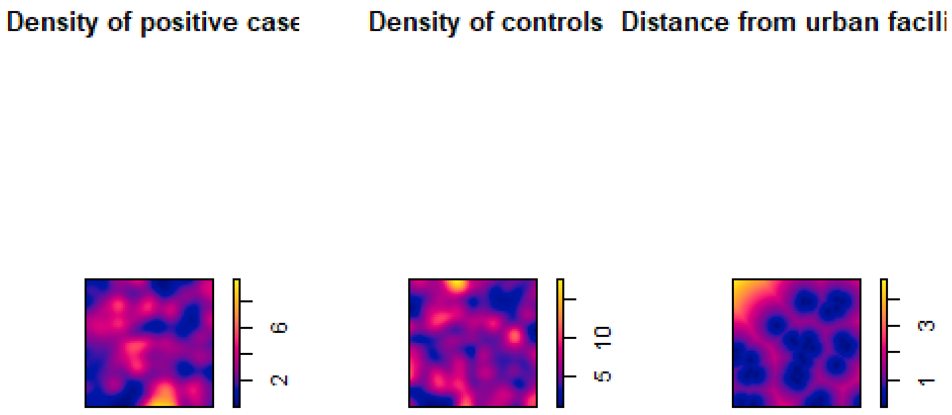

First order effect as a function of a spatial co-variate

Lastly, we calculate the non-parametric intensity estimation of both cases and controls as a function of distance from urban facilities as co variate.

Common-sense suggests that people tend to concentrate around urban facilities and viceversa. As such, it would be expected to find higher intensity of both cases and controls closer to urban facilities. If so, this would suggest that intensity of cases would be a function of the underlying pop. density, regardless of distance from urban facilities.

```
par(mfrow = c(1,2))

plot(rhohat(X_cases, X_facilities_dist), col = "red", main="Intensity of cases as a function of \ndistance from urban facilities", legend = FALSE)

Warning: In density.default(Zvalues, weights = if (horvitz) NULL else lambda/sum(lambda),
    bw = bwref, adjust = adjust, n = n, from = from, to = to,
    ..., warnWbw = FALSE) :
 extra argument 'warnWbw' will be disregarded

plot(rhohat(X_controls, X_facilities_dist), col = "blue", main="Intensity of controls as a function \nof distance from urban facilities", legend = FALSE)

Warning: In density.default(Zvalues, weights = if (horvitz) NULL else lambda/sum(lambda),
    bw = bwref, adjust = adjust, n = n, from = from, to = to,
    ..., warnWbw = FALSE) :
 extra argument 'warnWbw' will be disregarded
```

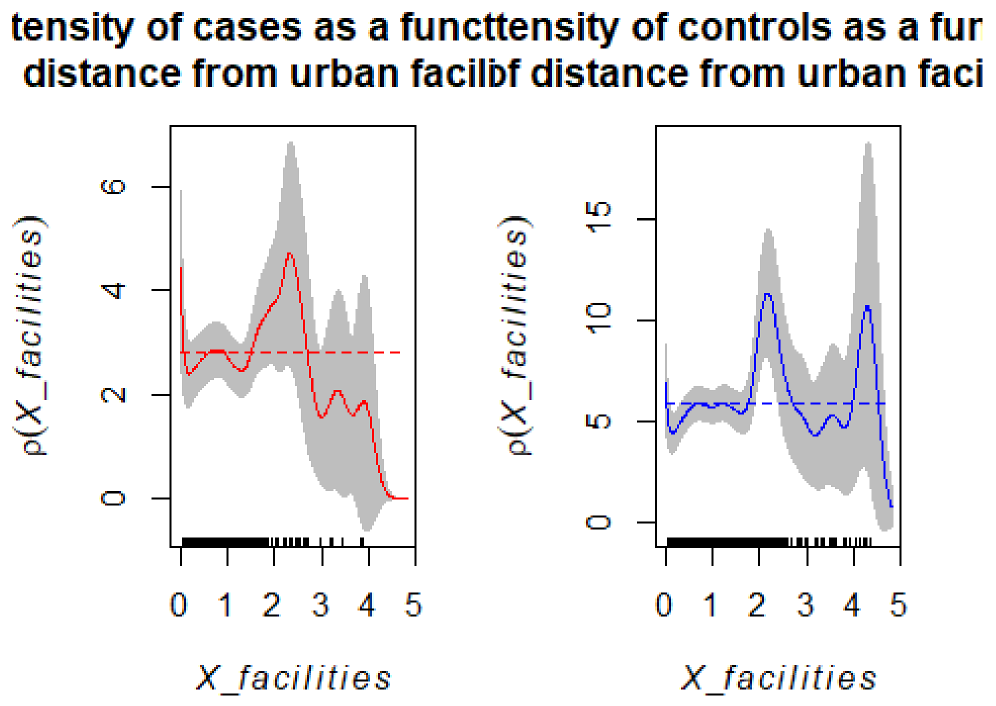

First order effect as a function of a pair of spatial co-variates

How to compare both intensity of cases and controls in relation to the distance from urban facilities? Since it is assumed that cases are proportional to population density.

The Smoothed Relative Density of Pairs of Co-variate Values (rho2hat) can be tried as well to assess the impact of both pop. density and urban facilities in the intensity of cases. This approach assumes that both of these co-variates are independent from each other, which seems unlikely.

```
plot(rho2hat(X_cases, D_controls, X_facilities_dist), main="Bivariate intensity of cases as a function of both \npopulation density & distance from urban facilities")
```

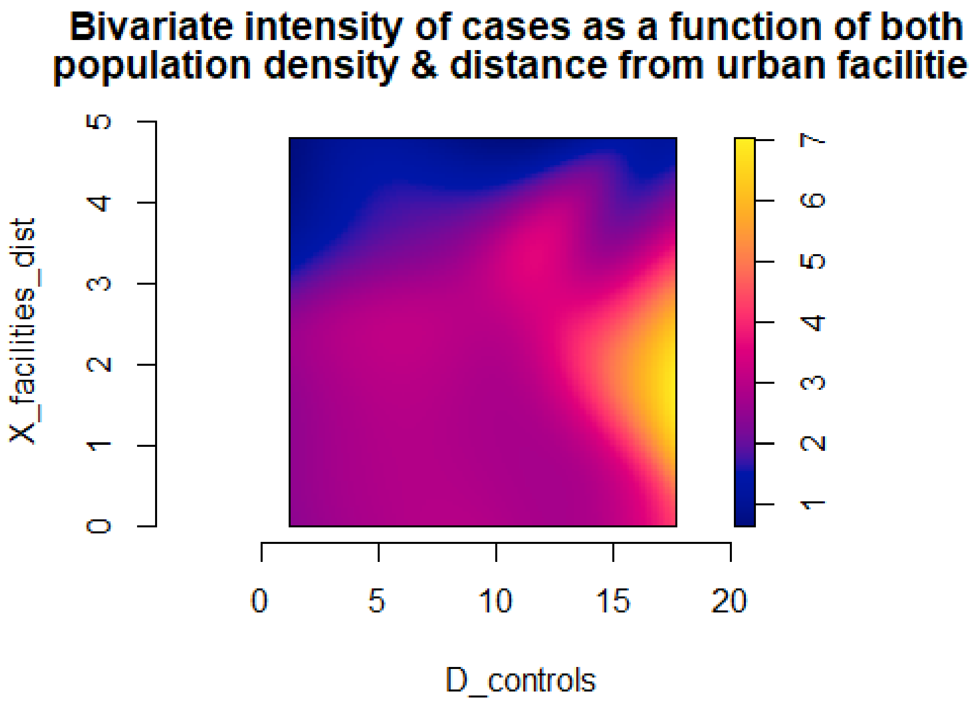

First order effect as a function of a spatial co-variate, from a given baseline

However, given that positive cases could be function of pop. density, a better approach consists on fitting a ppm model that accounts for the relative risk of cases, once taking controls into consideration, which in turn serves as a baseline for relative risk. This allows for exploring if, and how, the relative intensity of cases varies as a function of distance from urban facilities.

```
ppm_cases_controls <- ppm(X_cases, ~ D_controls)

plot(rhohat.ppm(ppm_cases_controls, X_facilities_dist), main="Relative Intensity (Relative Risk) of positive cases \nas a function of distance from urban facilities", legend = FALSE)
```

```
Warning: In density.default(Zvalues, weights = if (horvitz) NULL else
lambda/sum(lambda),
    bw = bwref, adjust = adjust, n = n, from = from, to = to,
    ..., warnWbw = FALSE) :
 extra argument 'warnWbw' will be disregarded

abline(h = 1, col="darkred", lwd=1, lty=2)
```

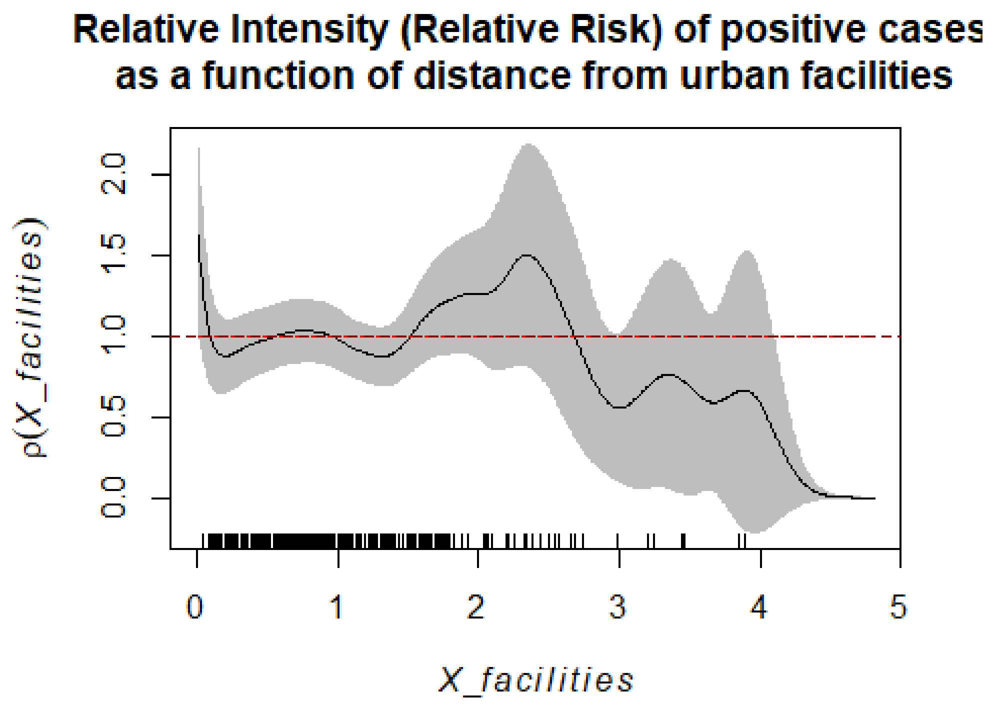

If the function oscillates below or above the baseline of 1, it would be an indication of the distance from urban facilities having some effect on the varying intensity of cases, once that population density has been accounted for. It is important to note that if the confidence envelope of the function (shaded area, at a 95% confidence level) encloses the baseline, it could be indication that there is no deviation from the baseline at all.

In order to assess the statistical significance of such deviations from the baseline, a Berman *Z1* test can be performed in order evaluate if the detected varying intensity is different of the variation that could be found under Complete Spatial Randomness (CSR).

```
plot((berman.test(ppm_cases_controls, X_facilities_dist)))
```

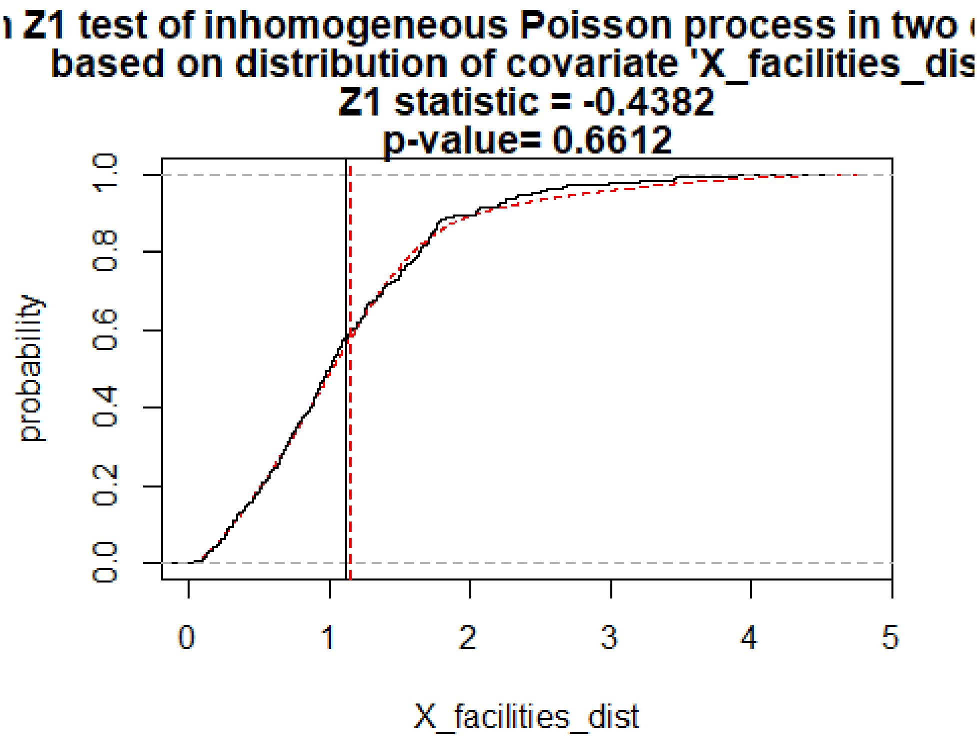

To exemplify what this test does, let's suppose we have a spatial co-variate *Z,* for instance, temperature, represented by the gradient color scheme; the lighter the color, the higher the value. For this co-variate, we also have its mean and standard deviation.

For the same study area, we have a spatial point pattern *X*, representing scorpion sting cases.

Are the values for temperature in the locations of *X* different from the values in a set of random points?

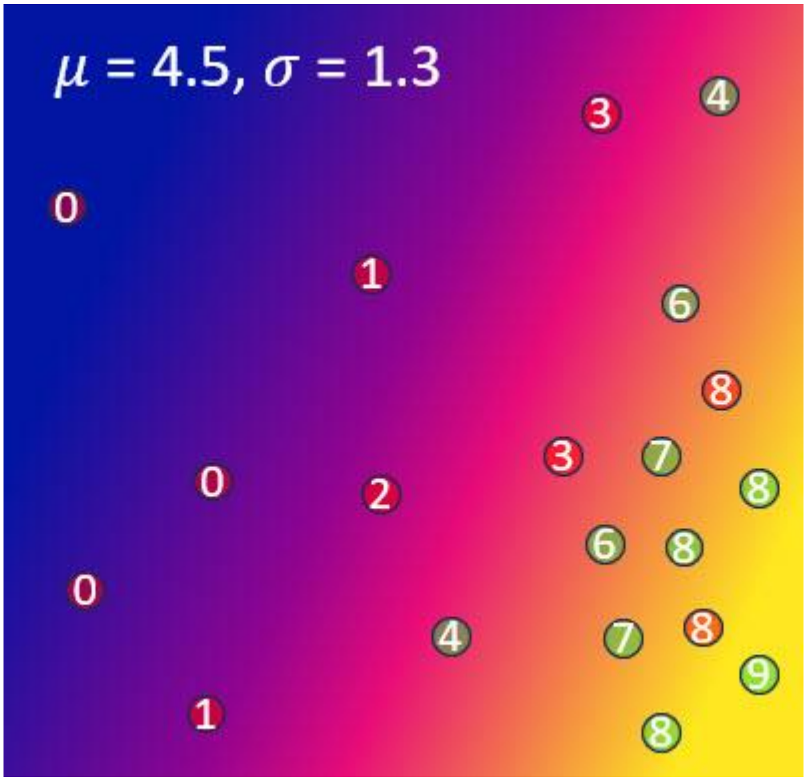

Example of a co-variate Z and a set of random points along the set of points in the point pattern X.

In order to test this, we perform a comparison between the values of the co-variate *Z* at locations *u* in a random set of points, and the values of the co-variate *Z* at locations *u* of the point pattern *X*.

Z(u) random locations	Cumulative probability Z(u) random locations	Z(u) at locations of point pattern X	Cumulative probability Z(u) at locations
0	0.000	4	0.060
0	0.000	4	0.119
0	0.000	6	0.209
1	0.038	6	0.299
1	0.077	7	0.403
2	0.154	7	0.507
3	0.269	8	0.627
3	0.385	8	0.746
8	0.692	8	0.866
8	1.000	9	1.000

With this data, it is possible to compute a Z test in order to assess if the differences between the co-variate values at locations u of the point pattern X are different from Complete Spatial Randomness.

$$z_1 = \frac{s - \mu}{\sigma},$$

$$z_1 = \frac{1.7 - 4.5}{1.3},$$

$$z_1 = -2.15,$$

$$p = 0.031.$$

A graph representing both cumulative functions illustrate such a difference. The closer the curves are, the more likely there are no differences when compared against CSR.

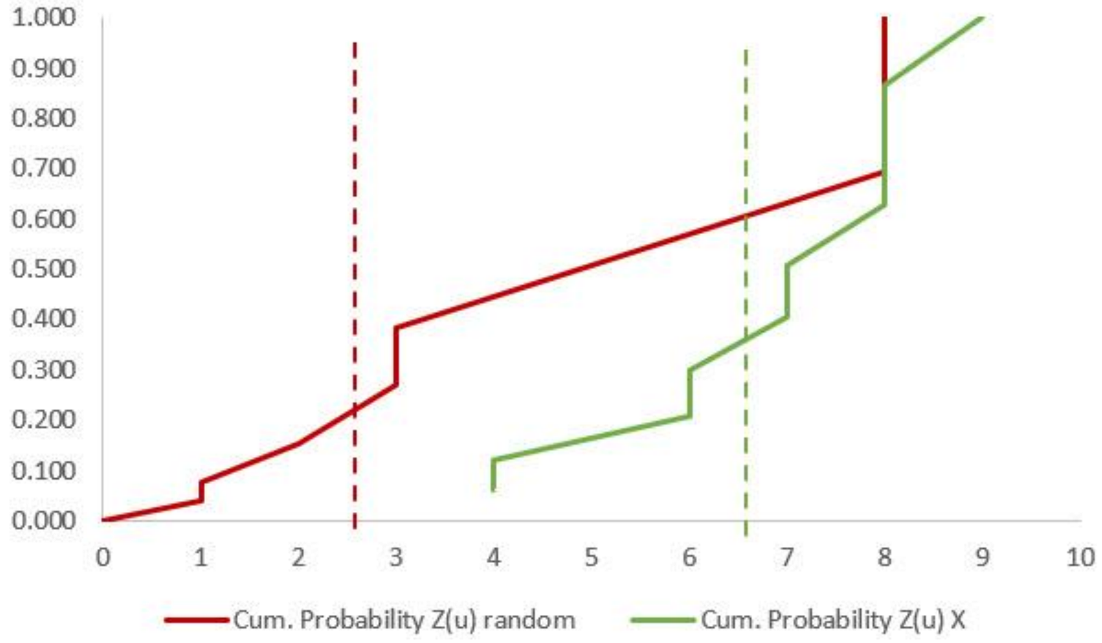

Baddeley, Adrian, Ya-Mei Chang, Yong Song, and Rolf Turner. 2012. "Nonparametric Estimation of the Dependence of a Spatial Point Process on Spatial Covariates." *Statistics and Its Interface* 5 (2): 221236. https://www.intlpress.com/site/pub/pages/journals/items/sii/content/vols/0005/0002/a007/.

Baddeley, Adrian, Ege Rubak, and Rolf Turner. 2015. *Spatial Point Patterns: Methodology and Applications with r*. CRC press. https://books.google.com/books?hl=es&lr=&id=rGbmCgAAQBAJ&oi=fnd&pg=PP1&d

q=point+pattern+analysis+covariates&ots=2zRKmJI5wu&sig=w88p6KI1uoF48iiM-su_06I3Nf0.

———. 2016. *Spatial Point Patterns. Methodology and Applications with r*. Florida: Chapman & Hall / CRC Interdiscplinary Statistics Series. https://doi.org/https://doi.org/10.1201/b19708.

Gatrell, Anthony C., Trevor C. Bailey, Peter J. Diggle, and Barry S. Rowlingson. 1996b. "Spatial Point Pattern Analysis and Its Application in Geographical Epidemiology." *Transactions of the Institute of British Geographers*, 256274. https://www.jstor.org/stable/622936.

———. 1996a. "Spatial Point Pattern Analysis and Its Application in Geographical Epidemiology." *Transactions of the Institute of British Geographers*, 256274. https://www.jstor.org/stable/622936.

González, Jonatan A., and Paula Moraga. 2023a. "Non-Parametric Analysis of Spatial and Spatio-Temporal Point Patterns." *The R Journal* 15 (1): 65–82. https://doi.org/10.32614/RJ-2023-025.

———. 2023b. "Non-Parametric Analysis of Spatial and Spatio-Temporal Point Patterns." *The R Journal* 15 (1): 65–82. https://doi.org/10.32614/RJ-2023-025.

Lambio, Christoph, Tillman Schmitz, Richard Elson, Jeffrey Butler, Alexandra Roth, Silke Feller, Nicolai Savaskan, and Tobia Lakes. 2023. "Exploring the Spatial Relative Risk of COVID-19 in Berlin-Neukölln." *International Journal of Environmental Research and Public Health* 20 (10): 5830. https://doi.org/10.3390/ijerph20105830.

Moraga, Paula. 2019. *Geospatial Health Data. Modeling and Visualization with r-INLA and Shiny*. Chapman & Hall / CRC Biostatistics Series. https://www.paulamoraga.com/book-geospatial/index.html.

O'Sullivan, David, and David Unwin. 2010. *Geographic Information Analysis*. 2nd ed. United States of America: John Wiley & Sons, Inc.
